# Intravenous iron supplementation in pulmonary hypertension groups 1 to 4

**DOI:** 10.7150/ijms.92904

**Published:** 2024-08-01

**Authors:** Leonie Biener, Josna Kohli, Vanessa Marggraf, Georg Nickenig, Dirk Skowasch, Carmen Pizarro

**Affiliations:** Department of Internal Medicine II - Pneumology/Cardiology, University Hospital Bonn, Bonn, Germany.

**Keywords:** Pulmonary hypertension, iron deficiency, iron supplementation

## Abstract

**Background:** Iron deficiency (ID) is common in patients with pulmonary arterial hypertension and has been associated with increased morbidity and mortality. We aimed to evaluate the therapeutic effects of iron supplementation in iron deficient patients with group 1 to 4 pulmonary hypertension (PH).

**Methods:** A total of 85 PH patients (mean age 69.8 ± 12.0 years, 56.5% female) were included in this prospective trial. Patients were screened for ID at baseline. PH patients with ID received intravenous iron supplementation (500-1000 mg ferric carboxymaltose). PH patients without ID served as control group. At baseline and 16-week follow up, six-minute walk test (6MWT), laboratory testing and echocardiography were performed. Additionally, World Health Organization (WHO) functional class, fatigue score and quality of life (QoL) by the SF-36 questionnaire were assessed.

**Results:** Overall, ID was present in 26.7% (n=8/30), 37.5% (n=9/24), 45.5% (n=10/22) and 44.4% (n=4/9) of patients in PH groups 1-4, respectively. In the total study population, iron restoration led to a significant mitigation of fatigue (p=0.01). However, 6MWT, WHO function class, NT-proBNP levels, QoL and right ventricular function did not change significantly. With regard to the underlying PH group, only PH group 3 patients experienced significant improvements in 6MWT distance (p=0.019), WHO functional class (p=0.017), fatigue (p=0.009) and some QoL domains, as compared to controls.

**Conclusions:** ID was common in PH groups 1 to 4. Though intravenous iron supplementation adequately restored iron status and improved fatigue throughout all patients, in the underlying PH groups treatment was accompanied by improvements in exercise capacity, WHO function class and fatigue only in group 3 PH.

## Introduction

Pulmonary hypertension (PH) represents a complex and heterogeneous disorder characterized by multiple underlying causes and pathophysiological processes. In accordance with the 2022 European Society of Cardiology (ESC) and European Respiratory Society (ERS) guidelines on PH, precapillary PH is defined by hemodynamic criteria assessed by right heart catheterization, with a mean pulmonary artery pressure >20 mmHg and a pulmonary vascular resistance > 2 Wood units 1. Besides the high mortality, PH patients - especially those with pulmonary arterial hypertension (PAH) - suffer from the effects of hypoxemia and right ventricular insufficiency, like dyspnoea, oedema and reduced physical performance. Morbidity is often amplified by cardiovascular and non-cardiovascular comorbidities 2,3.

In general, iron deficiency is associated with an impaired physical capacity, reduced quality of life (QoL) and increased mortality 4-7. In patients with chronic heart failure with reduced left ventricular ejection fraction (HFrEF), iron deficiency is a frequent comorbidity. In several randomized trials, the beneficial short- and longer-term effects of intravenous iron supplementation in iron-deficient HFrEF-patients have been demonstrated 8-10. In the aforementioned studies, iron supplementation improved exercise tolerance, clinical symptoms and QoL, and reduced hospitalizations and natriuretic peptide levels, often irrespectively of anaemia. Therefore, the European Society of Cardiology (ESC) on heart failure (HF) recommends regular monitoring for anaemia and iron deficiency; in iron-deficient HF patients, intravenous iron repletion with ferric carboxymaltose (FCM) should be considered (class IIa, level A) 11.

Likewise, iron deficiency is common in patients with PAH too. Its prevalence is high and ranges from 43 to 71 % 12-14. Similar to HFrEF-patients, iron deficiency in PAH has been associated with increased morbidity and mortality 14-16. In a study from 2014, Viethen et al. showed a significant increase in exercise capacity and QoL in 20 PAH patients with iron deficiency after a single infusion of FCM 17. According to the 2022 ESC/ERS guidelines on PH, periodical screening for iron deficiency is recommended in PAH patients. The recommendation for iron substitution in iron-deficiency anaemia was even revised and upvalued to a class I-recommendation. For iron deficiency without anaemia, the effect of iron substitution is less clear (class IIb, level C) 1.

Whether iron substitution is also beneficial in PH patients other than PAH, remains to be defined. Therefore, we aimed to evaluate the effect of iron substitution in PH groups 1 to 4 on exercise tolerance, N-terminal pro-brain natriuretic peptide (NT-proBNP) levels, right ventricular function, QoL and fatigue.

## Methods

This study was a prospective single centre study, investigating the effects of intravenous iron repletion in iron deficient patients with different types of pulmonary hypertension.

### Patient population

Between January 2016 and June 2018, a total of 85 patients aged ≥18 years participated in this prospectively conducted real-life study. All patients received outpatient treatment for PH at the Department of Pneumology, University Hospital Bonn (Bonn, Germany). Due to the exploratory nature of the study, we did not calculate a sample size but included all consecutive patients within the study period. Pulmonary hypertension was diagnosed by right heart catheterization. Patients with PAH, PH due to left heart disease, PH due to chronic lung disease and chronic thrombo-embolic pulmonary hypertension (CTEPH) were included. PAH and CTEPH patients were on stable targeted therapy for at least 3 months. Exclusion criteria for study participation comprised an existing oral or parenteral iron supplementation and clinical signs of acute inflammation at the time of evaluation. Other exclusion criteria included absence of or inability to provide study consent, as well as contraindications as per the iron carboxymaltose product information. Noteworthy contraindications comprise allergy to iron preparations, severe liver dysfunction or polycythaemia vera.

All patients underwent a standardized questionnaire-based clinical evaluation including World Health Organization function class (WHO-FC) assessment at baseline and at follow-up after 16 weeks. At these two points in time, laboratory and pulmonary function testing, transthoracic echocardiography and exercise capacity testing (6-minute walk test, 6MWT) were performed. Additionally, QoL by use of the SF-36 questionnaire and fatigue scores were assessed.

The study was approved by local ethics committee and complied with the Declaration of Helsinki (No 011/16). All patients gave their written informed consent prior to entry into the study.

### Study design

At baseline, patients were screened for iron deficiency, defined by a low transferrin saturation, defined by the local standard laboratory procedures (TSAT <16%), and ferritin levels of <150 µg/l in the absence of inflammation (high-sensitivity C-reactive protein (CRP) < 25 mg/l), for CRP values ≥ 25 mg/l ferritin values were not used due to probable alteration by systemic inflammation 17,18. Iron-deficient PH patients received intravenous iron supplementation with ferric carboxymaltose (FCM; Ferinject®, CSL Vifor, Glattbrugg, Switzerland). The iron demand was adjusted for body weight in accord with the prescribing information, and 500 - 1000 mg FCM were administered intravenously as a single dose. FCM was infused over 15 minutes in a solution of 100 ml 0.9% sodium chloride.

Patients without iron deficiency received no intervention and served as control group.

### Laboratory testing

All study participants underwent blood sampling at baseline and follow-up. Measurements encompassed iron status, including serum iron and ferritin level, transferrin saturation and soluble transferrin receptor (sTfR), as well as a complete blood cell count, high-sensitivity C-reactive protein (hs-CRP) and NT-proBNP levels.

Anaemia was defined according to WHO criteria by a haemoglobin concentration < 13 g/dl in men and a haemoglobin concentration < 12 g/dl in women.

### Six-minute walk test

Exercise capacity was assessed by standardized six-minute walk test (6MWT) according to the ATS guidelines 19.

### Assessment of quality of life and fatigue

Quality of life was measured by use of the SF-36 questionnaire, a scale that covers four physical and four mental health domains with a scale of 0-100 points each. Higher scores indicate a better QoL 20.

In addition, patients completed the Fatigue Score, a numeric rating scale from 1-10. The higher the score, the higher the fatigue 21.

### Pulmonary function testing and capillary blood gas analysis

Pulmonary function testing and capillary blood gas analysis were conducted in a routine clinical setting.

Pulmonary function testing comprised spirometry, bodyplethysmography and determination of diffusion capacity for carbon monoxide (Bodyplethysmograph Jaeger©, Alveo-Diffusionstest Jaeger©, Wuppertal, Germany).

Capillary blood gas analysis was performed with a sample collected from the earlobe.

### Transthoracic echocardiography

A complete 2D transthoracic echocardiography study was conducted by experienced cardiac sonographers at the University Hospital´s cardiological unit at baseline and follow-up. For echocardiography, a 2.5 MHz phased-array transducer and a standard ultrasound scanner were used (iE 33, Philips Medical Systems, Koninklijke NV, Hamburg, Germany), in conformity with the recommendations of the American Society of Echocardiography 22. The assessment was focused on parameters of right ventricular function. Therefore, measurements of the systolic pulmonal arterial pressure (sPAP) and tricuspid annular plane systolic excursion (TAPSE) were obtained.

### Statistical analysis

Categorical data are presented as percentages. Continuous variables are presented as mean ± one standard deviation and were analysed at baseline by a two-sided unpaired Student's *t*-test. Homogeneity of variance was assessed by use of Levene's test. In case of unequal variances, Welch's *t*-test was performed. At follow up, in the case of paired data, paired *t*-test was applied and effect size was calculated by Cohen's *d*. Categorical variables were compared by Fisher's exact test or the chi-square test for trend, as appropriate. For analyses of subgroups (n≤30), non-parametric tests were used: Wilcoxon signed-rank test in the case of paired data and Mann-Whitney U test in the case of unpaired data. A p-value < 0.05, resulting of 2-tailed tests, was considered threshold for statistical significance. Statistical analyses were performed using SPSS Statistics 26 software (IBM, Armonk, NY, USA).

## Results

### Results in the total study population

#### Clinical characteristics at baseline of the total study population

Demographic features and clinical data of study participants at baseline are displayed in Table 1. 85 patients were included in the study. The study population exhibited a slight female predominance (56.5%, n=48) and a mean age of 69.8 ± 12.0 years. Baseline laboratory testing revealed that 31 PH patients had an iron deficiency and thus received iron substitution (henceforth referred to as intervention group). 54 PH patients without iron deficiency served as control group.

As displayed in Table 1, there were no significant differences between the intervention and the control group with regard to age, sex, comorbidities or medication. None of the parameters obtained by pulmonary function testing, capillary blood gas analysis, echocardiography and fatigue assessment differed significantly between intervention and control group.

#### Iron deficiency and iron deficiency anaemia

**Figure 1:**
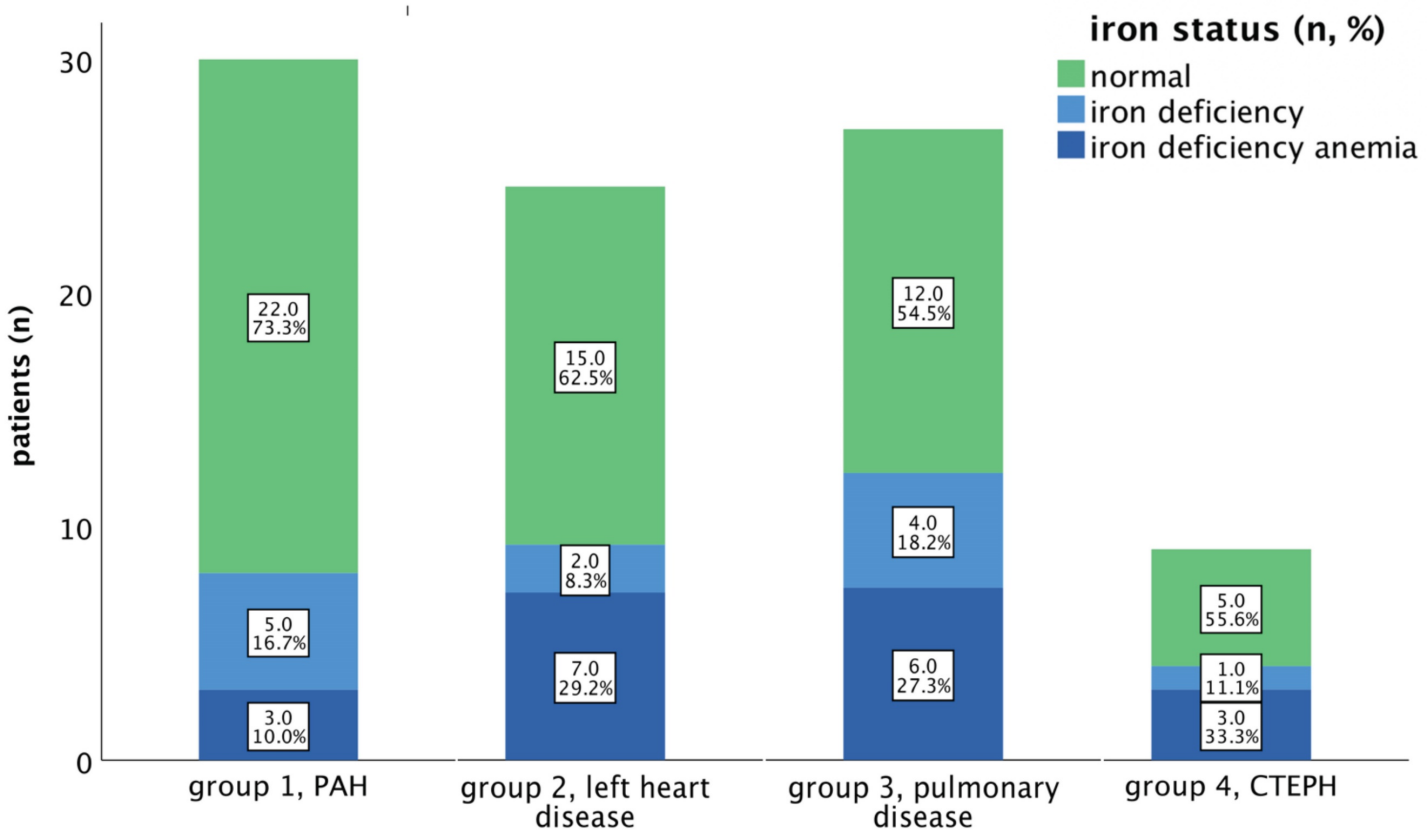
Prevalence of iron deficiency (without anaemia) and iron deficiency anaemia in PH groups 1-4. Notes: Percentages refer to the cumulative percentages of the respective group. Abbreviations: CTEPH: chronic thromboembolic pulmonary hypertension. PAH: pulmonary arterial hypertension.

In our total study group, 31 patients (36.5%) exhibited iron deficiency (ID), of which 19 patients (22.4 % of all patients) were diagnosed with an iron deficiency anaemia (IDA). The prevalence rates of ID (without anaemia) and IDA within the respective subgroups are illustrated in Figure 1.

Among our PAH cohort, the n=8; 26.7% had an iron deficiency, of which n=3; 10.0% had IDA. In our PH group 2 study cohort, ID was identified in 9 out of 24 patients (37.5%); while IDA was observed in 7 out of 24 patients (29.2%).

Regarding PH group 3 and 4, ID was present in 10 out of 22 (45.5%) and 4 out of 9 patients, while IDA was observed in 6 out of 22 patients (27.3%) and 3 out of 9 patients (33.3%), respectively (depicted in Figure 1).

#### Laboratory testing

From baseline to follow-up, serum iron and ferritin levels as well as transferrin saturation improved significantly upon intravenous iron substitution. Haemoglobin concentration increased by 0.42 g/dl, but not significantly. Haematocrit, erythrocyte count and soluble transferrin receptor did not change in the intervention group but worsened slightly but significantly in the control group (Table 2).

Despite the improvements in iron status, plasma NT-proBNP concentration did not change significantly from baseline to follow-up, neither in the intervention, nor in the control group (Table 3).

#### Exercise capacity and WHO functional class

The 6MWT-distance and WHO function class did not change significantly from baseline to follow up (Table 3).

The percentage of patients in WHO-FC I or II changed from 51.6% to 54.8% (p=0.66) in the intervention group, and from 57.4% to 48.1% in controls (p=0.18).

#### Quality of life and fatigue

Among all patients, there were no meaningful changes in the SF-36 score domains, except mental health score (MH) which deteriorated significantly in both intervention and control group (p<0.001 each), with no significant difference between the delta from baseline to follow up (depicted in Supplementary Figure S1).

Fatigue significantly decreased after iron substitution (5.6 ± 2.1 to 4.5 ± 2.0, p=0.01), whereas it did not change substantially in controls (5.1 ± 2.7 to 5.4 ± 2.9, p=0.18) (Table 3).

#### Pulmonary function testing and echocardiography

As given in Table 3, forced expiratory volume in one second (FEV1) and forced vital capacity (FVC) improved significantly in the intervention group (FEV1 +0.07 L, 95%CI: +0.01 - +0.1, p=0.02; FVC +0.2 L, 95%CI: +0.01 - +0.3, p=0.04), as did FVC in the control group (p=0.03).

Echocardiographic measurements did not change significantly from baseline to follow-up, neither in the intervention, nor in the control group (Table 3).

### Results in PH groups 1-4

With regard to PH aetiology, 35.3% (n=30), 28.2% (n=24), 25.9% (n=22) and 10.6% (n=9) of study participants belonged to PH groups 1-4, respectively. With the exception of iron status parameters, baseline characteristics did not differ between iron-deficient and iron-sufficient patients in none of the four PH groups (Supplementary Tables 4-7).

Subgroup analysis was performed to detect potential benefits of iron substitution, depending on the underlying PH group. Due to the rather small number of patients in Group 4, we did not conduct subgroup analysis for this group.

**Figure 2:**
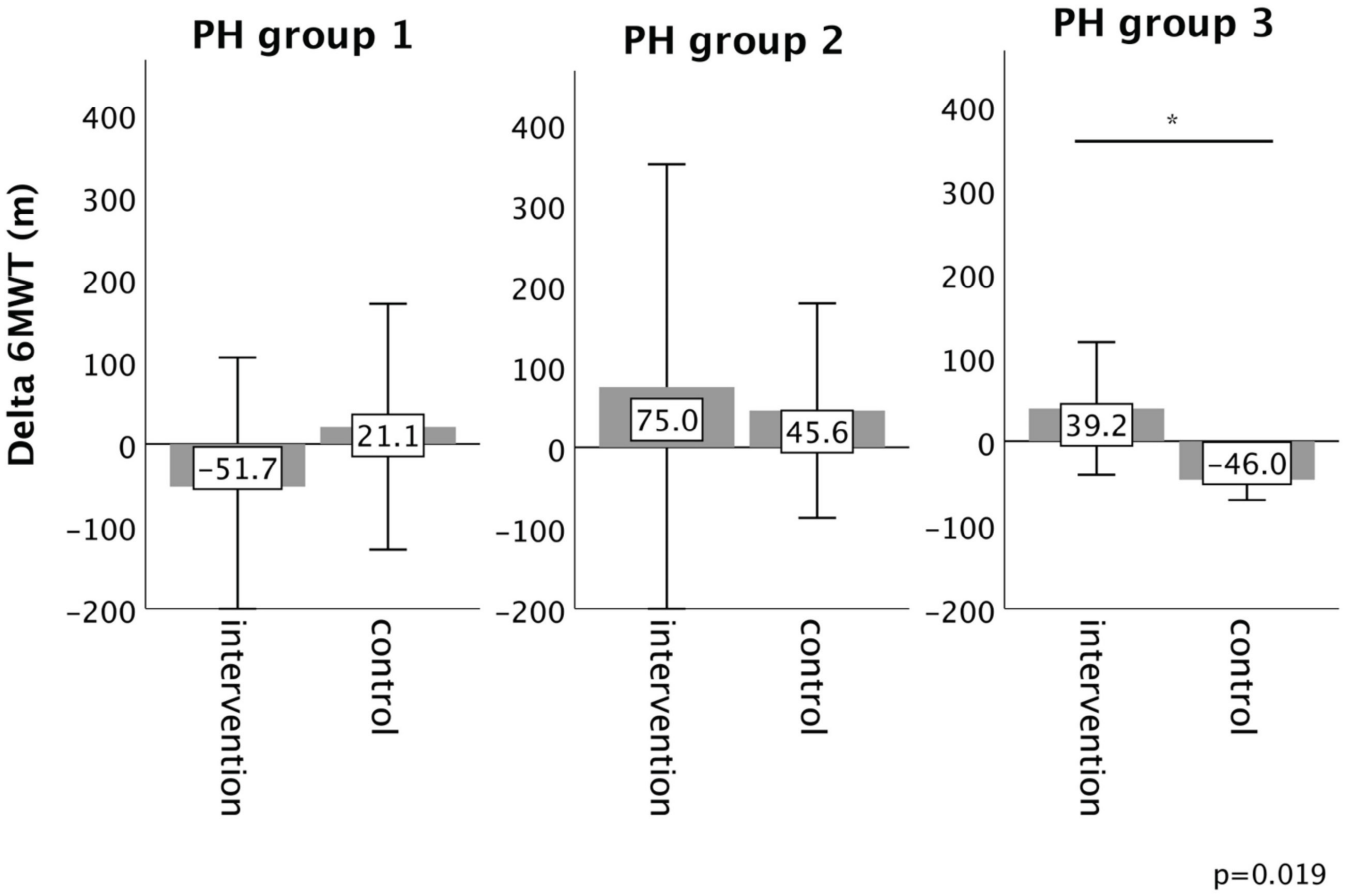
Changes in six-minute walk test distance (6MWT) from baseline to 16-week follow up in the intervention group (iron deficient PH patients) and controls, stratified by the underlying PH group 1-3. Notes: values represent mean ± standard deviation. P-values compare changes in controls and intervention group (Mann-Whitney U test). Asterisks indicate a significant p-value <0.05. Abbreviations: PH: pulmonary hypertension. 6MWT: six-minute walk test.

**Figure 3:**
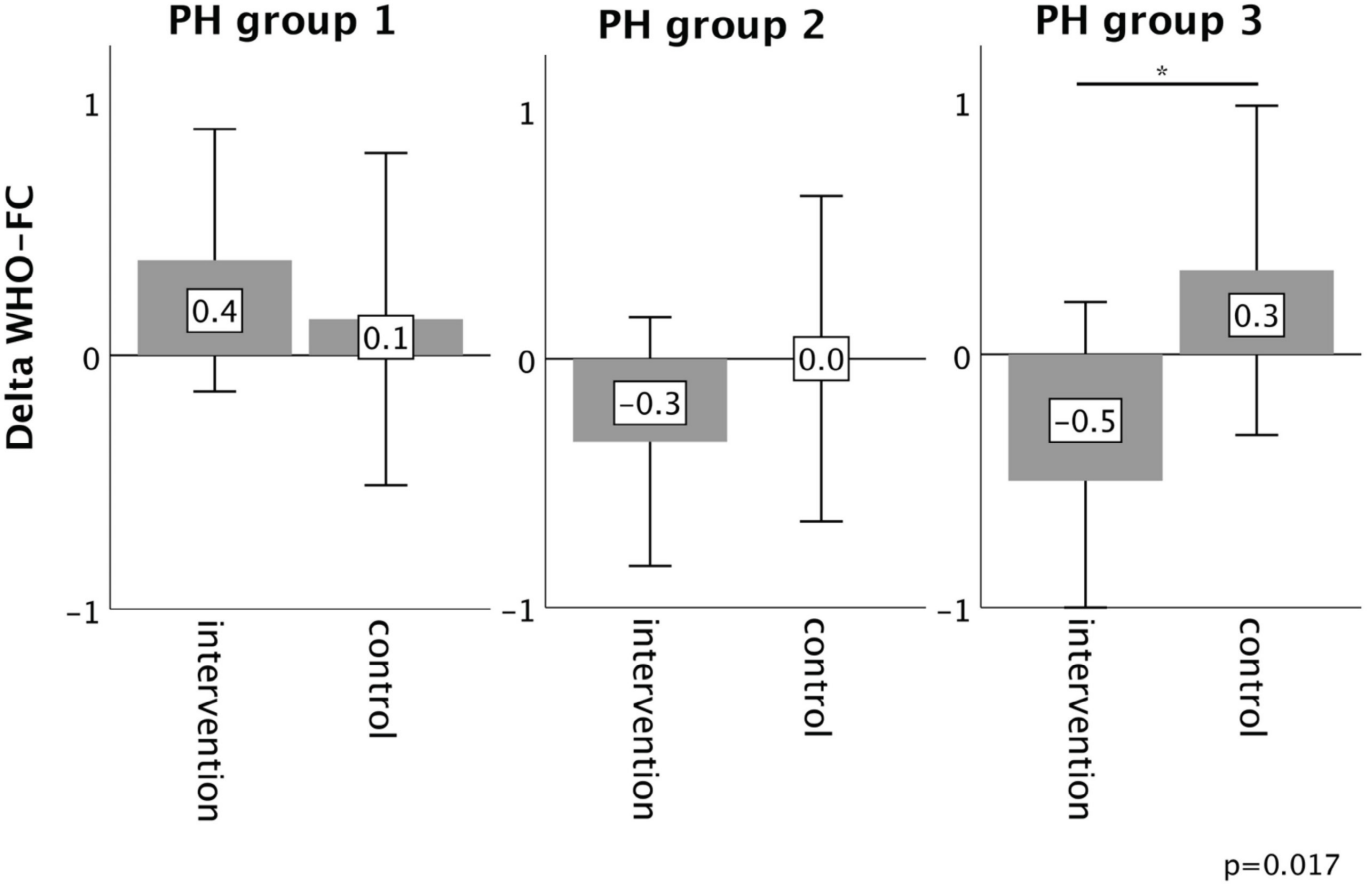
Changes in WHO function class from baseline to 16-week follow up in the intervention group (iron deficient PH patients) and controls, stratified by the underlying PH group 1-3. Notes: values represent mean ± standard deviation. P-values compare changes in controls and intervention group (Mann-Whitney U test). Asterisks indicate a significant p-value <0.05. Abbreviations: PH: pulmonary hypertension. WHO-FC: WHO function class.

**Figure 4:**
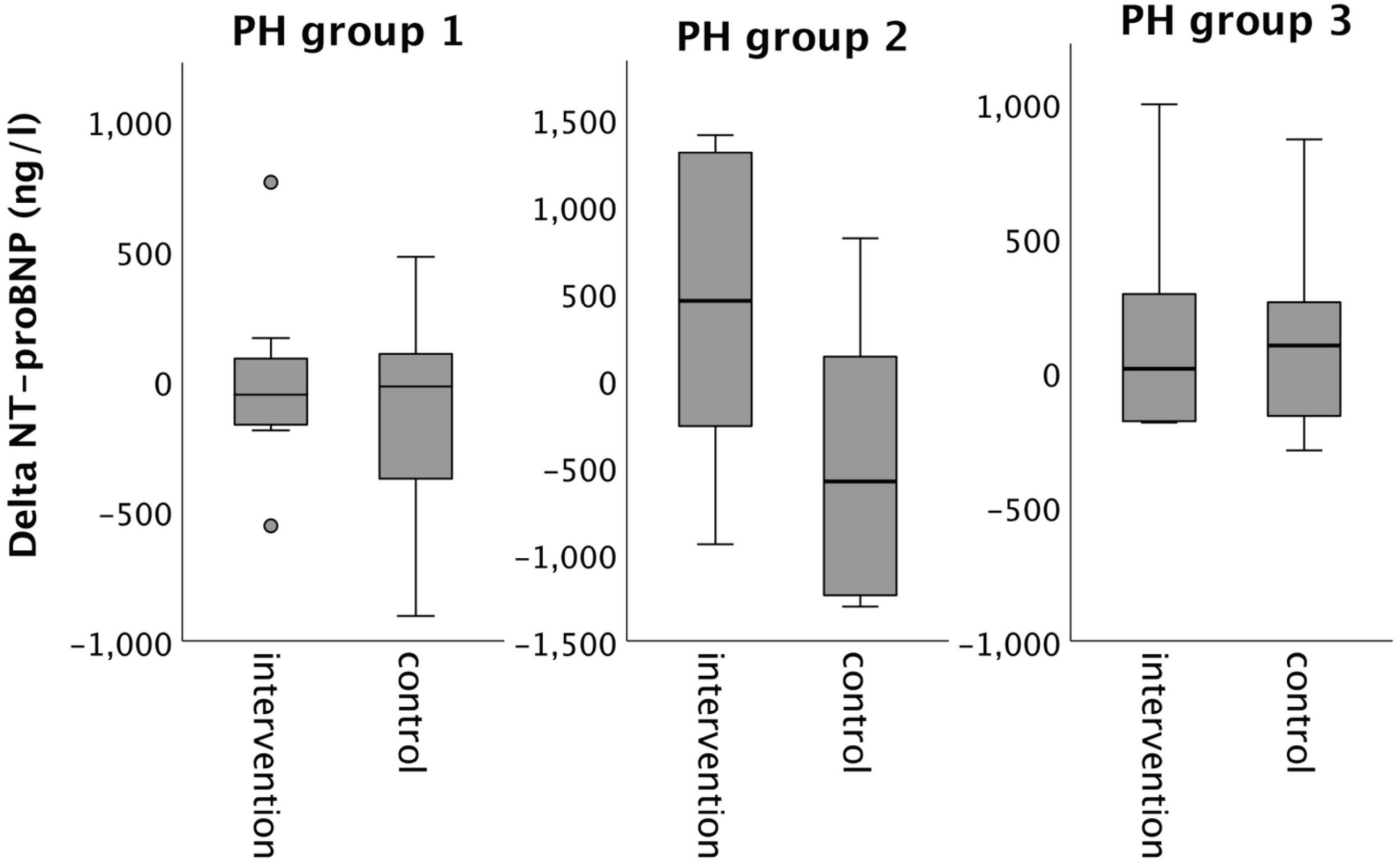
Changes in nt-pro-BNP from baseline to 16-week follow up in the intervention group (iron deficient PH patients) and controls, stratified by the underlying PH group 1-3. Notes: values represent mean ± standard deviation. P-values compare changes in controls and intervention group (Mann-Whitney U test). Asterisks indicate a significant p-value <0.05. Abbreviations: NT-proBNP: N-terminal pro Brain Natriuretic Peptide. PH: pulmonary hypertension.

**Figure 5:**
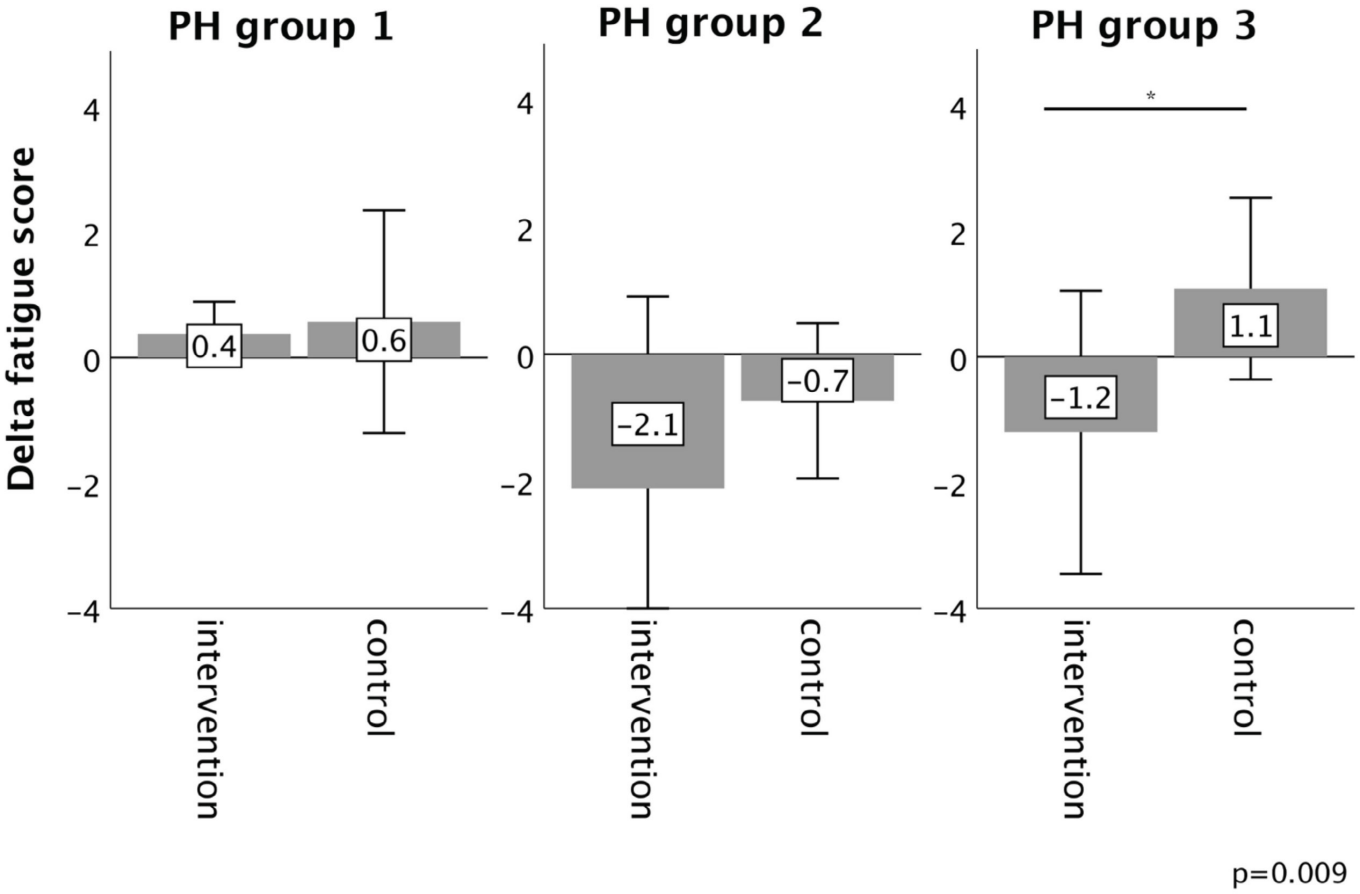
Changes in fatigue score from baseline to 16-week follow up in the intervention group (iron deficient PH patients) and controls, stratified by the underlying PH group 1-3. Notes: values represent mean ± standard deviation. P-values compare changes in controls and intervention group (Mann-Whitney U test). Asterisks indicate a significant p-value <0.05. Abbreviations: PH: pulmonary hypertension.

**Figure 6:**
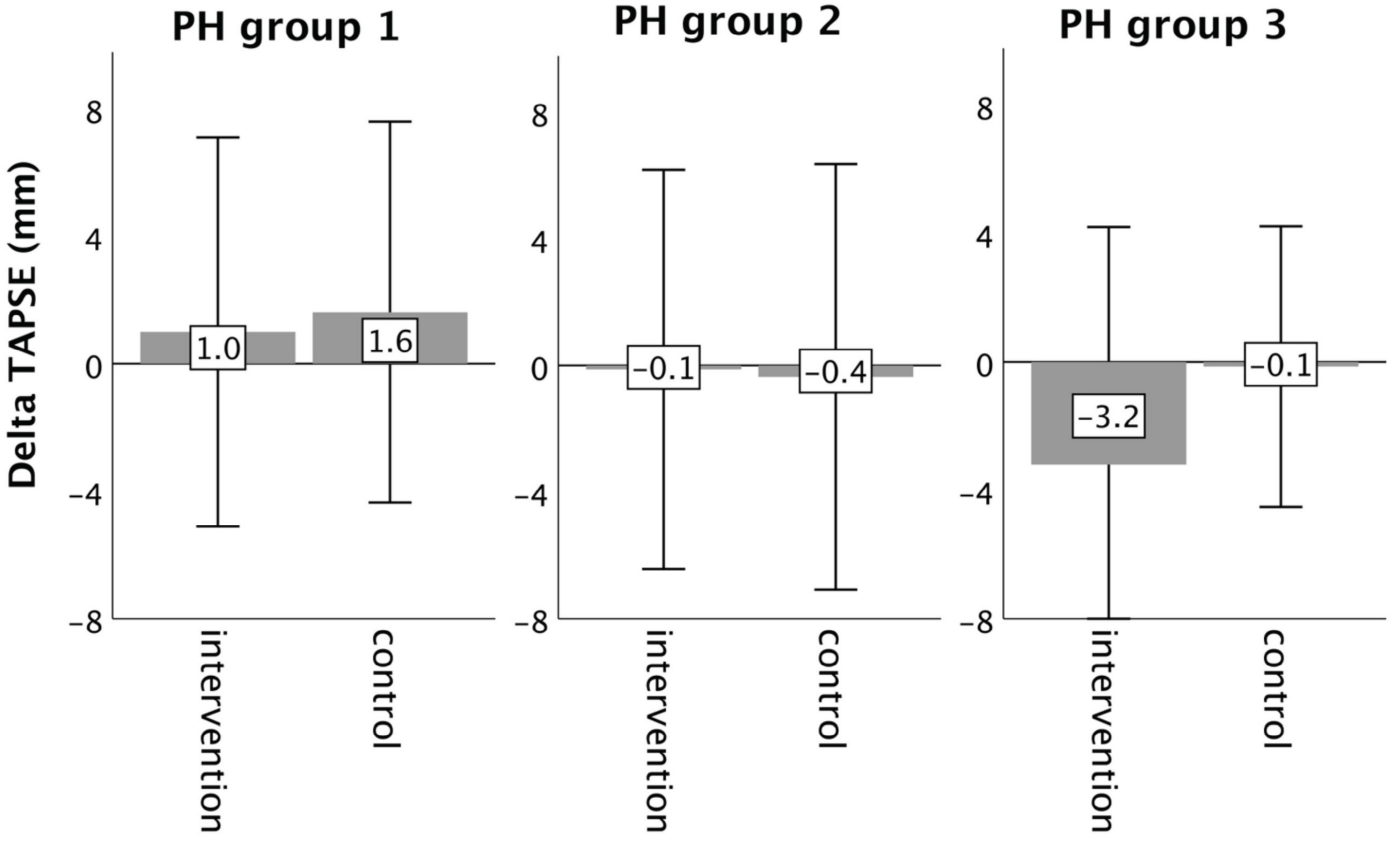
Changes in right ventricular function (TAPSE) from baseline to 16-week follow up in the intervention group (iron deficient PH patients) and controls, stratified by the underlying PH group 1-3. Notes: values represent mean ± standard deviation. P-values compare changes in controls and intervention group (Mann-Whitney U test). Asterisks indicate a significant p-value <0.05. Abbreviations: PH: pulmonary hypertension. TAPSE: tricuspid annular plane systolic excursion.

In PH group 1 and 2, changes in 6MWT, WHO functional class, NT-proBNP levels, fatigue and right ventricular function in the intervention group did not significantly differ from those experienced in the respective control group, as depicted in Figures 2-6. However, in PH group 3, iron substitution was associated with significant improvements in 6MWT distance (p=0.019), WHO functional class (p=0.017), and fatigue (p=0.009), as compared to controls (displayed as bar graphs with means ± standard deviation (SD) for reasons of comprehensibility in Figures 2-3, 5 and 6). In PH group 3 (intervention) six patients improved WHO-FC, of which four moved from class III to II and two from class II to I, three remained in the same WHO-FC, one patient worsened from class I to II. In the PH group 3 controls, only one patient improved from WHO-FC III to II, four worsened from class II to class III and one from class III to IV, while six remained in the same WHO-FC.

Quality of life (QoL) as assessed by SF-36 score showed a significant deterioration in “general health” as a physical measure of the SF-36 score (p=0.008), and “mental health” as a mental health measure in the group 3 control patients (p<0.001), but not in group 3 patients who received iron supplementation. The delta in the change from baseline to follow-up differed significantly in both score domains between the intervention and control group (p=0.002 respectively p=0.049). “Vitality” increased in group 3 patients with iron supplementation, but not in control group. For the other score domains and patient groups, results were inconsistent. SF-36 score data are displayed at Supplementary Figure S1.

## Discussion

This is the first study to investigate the therapeutic effect of iron supplementation in iron-deficient patients of different PH aetiologies.

The main findings are:

1. The prevalence of iron deficiency (anemia) in our cohort of patients in PH groups 2-4 ranged from 37.5% (group 2) to 45.5% (group 3) and was higher than its prevalence in our PAH collective (26.7%).

2. At entry, iron-deficient PH patients exhibited no differences in WHO function capacity, physical performance or NT-proBNP levels as compared to PH patients without iron deficiency.

3. Overall, iron supplementation repleted iron stores, but had only beneficial effects on patients' exercise response, functional class and fatigue in group 3 PH.

In PAH, several previous studies have reported that iron restoration improves symptoms, physical performance and mortality 14,23,24. The underlying mechanisms are diverse and include a hepcidin-dependent iron malabsorption with elevated hepcidin levels inhibiting gastrointestinal iron uptake 25. These trials were limited due to their uncontrolled, open-label study design. In 2021, Howard and colleagues reported two randomized, double-blind studies on intravenous iron supplementation versus placebo in iron-deficient PAH patients without anaemia 26. Iron infusion had no measurable effect on exercise testing, NT-proBNP levels or haemodynamics. These findings temper the expectation of a generic benefit accompanying iron restoration in PAH. In line with Howard et al., iron supplementation in our PAH cohort did not impact exercise response or symptomatology. The notion of a mechanistic link between iron deficiency and PAH remains controversial. Ulrich et al. used a Mendelian randomization approach to assess causality between red cell distribution width (a corelate of iron homeostasis) and PAH 27. They found no causal link between both of them and deduced that study results on iron supplementation in PAH should be interpreted cautiously.

In left heart failure, iron deficiency is common with a prevalence up to 55% 28,29. Moreover, it impacts patients' frailty, irrespective of anaemia. On the other hand, a growing body of evidence indicates that restoring iron levels significantly improves physical performance, quality of life and functional capacity in chronic left heart failure 8,10. However, none of these trials provided a subgroup analysis on the impact of concomitant PH in left heart disease. In our PH group 2 study cohort, the prevalence iron deficiency and - anaemia was much lower. Our PH group 2 study cohort was not homogenous, as the underlying left heart diseases comprised HFrEF, as well as HF with preserved ejection fraction and valvular heart diseases. Independent of concomitant PH, the significance of iron supplementation in both last-mentioned left heart disease entities remains to be defined and is subject of research in ongoing trials (e.g., FAIR-HFpEF).

Within group 3 PH, 10 out of 22 patients (45.5%) were iron deficient; iron deficiency anaemia occurred in 6 of those patients (27.3%). Chronic obstructive pulmonary disease (COPD) was the most common underlying disease (n=18), the remaining 4 patients suffered from lung fibrosis.

Overall, data on iron status in group 3 PH are sparse: Tatah and colleagues reported a prevalence of 53% of iron deficiency within group 3 patients 30. When compared to non-deficient patients, iron deficiency did not impact 6MWT distance nor right ventricular function, as assessed by echocardiography. Consistent with these findings, we found no differences in exercise capacity (6MWT) between iron deficient and iron replete group 3 PH patients at baseline. However, at 16-week follow up, the 6MWT distance increased after iron administration, whereas it deteriorated in the control group. These changes were accompanied by a significant improvement in WHO function class and gain in QoL, and exceeded the minimal clinical important difference (MCID) for the 6MWT. The mechanisms on how iron substitution may have impacted the exercise response only in this PH group, but not in any of the other here studied PH groups, may be due to the importance of iron for hypoxic vasoconstriction, the main driver of PH in chronic lung diseases. Iron availability modifies pulmonary vasoconstrictive response to alveolar hypoxia: iron repletion mitigates this response, whereas iron deficiency intensifies it 31. That may cause the particular vulnerability of patients with group 3 PH to iron status derangements. However, the underlying mechanisms are likely to be more complex and not entirely explicable.

Vinke and colleagues evaluated the prevalence of micronutrient deficiencies in CTEPH patients 32. In newly diagnosed CTEPH, 21% of patients were iron deficient; in patients under CTEPH-treatment for 1.5 years, the prevalence was similar (20%). In the entire CTEPH collective, a significant positive correlation was found between the transferrin saturation and the 6MWT distance. With regard to WHO functional class, iron deficient patients tended to have higher WHO function class and thus lower functional capacity, although the difference was not statistically significant. However, data on iron restoration in iron deficient CTEPH patients are missing by now. In our CTEPH study cohort, the prevalence of iron deficiency was even higher than the one reported by Vinke et al. (44.4%). At baseline, iron deficient CTEPH patients exhibited no differences in exercise capacity (6MWT distance) or WHO functional class, when compared to iron sufficient CTEPH patients, comparable to the other subgroups results. To meaningfully investigate this subgroup in terms of responsiveness to iron substitution, however, studies with larger patient cohorts are necessary.

There are several limitations that should be addressed. Maybe the most significant limitation pertains to the heterogeneity of the patient population. Given the exploratory and real-world design of our study, we imposed only minimal exclusion criteria. Findings must be interpreted with caution, as the whole patient group encompasses all four pulmonary hypertension (PH) groups. Hence, we conducted subgroup analysis. However, due to the subdivision into four subgroups, the sample sizes within each group are relatively small. Further studies with larger patient cohorts are necessary to validate the hypotheses generated.

Secondly, the uncontrolled and unblinded single-centre study design in small populations of PH groups 1-4 restricts generalizability of study results. Thirdly, we assessed iron deficiency by measurement of transferrin saturation. This biochemical indicator can be impacted by inflammation as it potentially lowers serum transferrin saturation levels. To mitigate this effect, we excluded PH patients with clinical and biochemical signs of acute inflammation (hs-CRP > 25 mg/l) from study participation. However and contrarily to the ESC guidelines on heart failure 11, European ESC/ERS guidelines on PH did not clarify which clinically available parameter accurately define iron deficiency in PH at the time of our study conduction 33. The 2022 ESC/ERS guidelines on PHA include the same definition if ID as for heart failure (ferritin <100 µg/l or 100-299 µg/l and transferrin saturation <20%) 1. As the new definition is more inclusive, most likely more patients would have been diagnosed with iron deficiency and treated accordingly. Therefore, according to the new definition, our control group may include patients with iron deficiency who did not receive treatment. This does not alter the outcome that patients with PH and iron deficiency did not benefit from therapy during our observation period (or that group 3 patients did benefit, respectively). However, to confirm the thesis, larger studies based on this definition of ID are necessary.

Additionally, patients were followed up at 16 weeks of iron infusion. The interval might have been too short-termed to detect signals of benefit of iron supplementation. Nonetheless, we observed significant improvements in iron levels and repleted iron stores at 16 weeks of iron supplementation. Noteworthy, iron status deteriorated significantly in control patients. It emphasizes the necessity for regular reassessment of iron homeostasis in PH, as recommended by current ESC/ERS guidelines 1.

In conclusion, iron deficiency was common in PH groups 1-4 and intravenous iron supplementation adequately restored iron levels at 16 weeks. In the total study population, these changes were accompanied by improvements in fatigue. Only in group 3 PH iron restoration additionally provided benefits in exercise capacity and WHO function class. This trial mitigates the potential benefits of such a treatment in other PH groups beyond group 3 but sets the stage for lager studies examining iron therapy in the broad range of PH subgroups.

## Supplementary Material

Supplementary figures and tables.

## Figures and Tables

**Table 1:** Baseline characteristics of total study population (n = 85)

	Intervention (n = 31)	Control (n = 54)	p-value
Age [years]	69.1 ± 12	70.2 ± 12	0.67
Male	13 (41.9 %)	24 (44.4 %)	0.50^C^
Aetiology of PH			0.51^ F^
- PAH	8 (25.8 %)	22 (40.7 %)	
- PH-LHD	9 (29.0 %)	15 (27.8 %)	
- PH-CLD	10 (32.3 %)	12 (22.2 %)	
- CTEPH	4 (12.9 %)	5 (9.3%)	
Long-term oxygen therapy [n]	9 (29.0 %)	19 (35.2 %)	0.37^C^
Long-term oxygen therapy [l/min]	1.3 ± 1.7	1.2 ± 2.5	0.83
WHO function class			0.63^F^
- I	3 (9.7 %)	9 (16.7 %)	
- II	13 (41.9 %)	22 (40.7 %)	
- III	14 (45.2 %)	19 (35.2 %)	
- IV	1 (3.2 %)	4 (7.4 %)	
Fatigue score	5.6 ± 2.1	5.1 ± 2.7	0.38
Medication			
PAH Medication			0.76^F^
- ERA	3 (9.7 %)	7 (13.0 %)	
- PDE5i	4 (12.9 %)	2 (3.7 %)	
- sGC stimulator	1 (3.2 %)	1 (1.9 %)	
- ERA + PDE5i	6 (19.4 %)	11 (20.4 %)	
- ERA + sGC stimulator	0 (0.0 %)	3 (5.6 %)	
- ERA + PDE5i + prostacyclin analogue	0 (0.0 %)	1 (1.9 %)	
Other Medication			
- Diuretics	24 (77.4 %)	43 (79.6 %)	0.51^C^
- Platelet aggregation inhibitor	13 (41.9 %)	14 (25.9 %)	0.10^C^
- Oral anticoagulation	14 (45.2 %)	25 (46.3 %)	0.65^C^
- ACE inhibitor	14 (45.2 %)	26 (48.1 %)	0.83^C^
- AT1 receptor antagonist	6 (19.4 %)	13 (24.1 %)	0.79^C^
- Beta blocker	20 (64.5 %)	29 (53.7 %)	0.37^C^
- Calcium-Antagonist	5 (16.1 %)	14 (25.9 %)	0.42^C^
- Cardiac glycoside	1 (3.2 %)	4 (7.4 %)	0.65^F^
- Statin	17 (54.8 %)	24 (44.4 %)	0.38^C^
6-minute walk test			
Walk distance [m]	285.8 ± 123.8	327.6 ± 136.6	0.86
Laboratory assessment			
Hs-CRP [mg/l]	7.2 ± 6.9	4.6 ± 4.5	0.07^+^
NT-proBNP [ng/l]	2448.5 ± 3811	2647.3 ± 3835	0.72
Creatinine [mg/dl]	1.3 ± 0.6	1.2 ± 0.5	0.38
ALT [U/l]	26.6 ± 13	28.3 ± 17	0.63
Gamma-GT [U/l]	68 ± 75	94.3 ± 107	0.24
LDH [U/l]	249 ± 91	257.1 ± 72	0.65
Pulmonary function testing			
FEV1 [L]	1.6 ± 1	1.6 ± 1	0.73
FEV1 [% pred]	62.1 ± 22	64.1 ± 24	0.71
FVC [L]	2.1 ± 1	2.4 ± 1	0.17
FVC [% pred]	66 ± 20	71.5 ± 20	0.23
TLC [% pred]	90.4 ± 28	93 ± 24	0.66
RV [% pred]	137.4 ± 70	142.4 ± 53	0.71
Rtot [% pred]	160.3 ± 119	161.5 ± 166	0.97
DLCO [% pred]	38.8 ± 16	47.9 ± 19	0.08
Capillary blood gas analysis			
pO2 [mmHg]	66.6 ± 13	69 ± 12	0.41
pCO2 [mmHg]	36 ± 7	35 ± 7	0.55
Echocardiography and Electrocardiogram			
Heart rate [n/min]	76.6 ± 16	74.3 ± 13	0.50
LVEF [%]	56.8 ± 13	56.5 ± 15	0.94
sPAP [mmHg + ZVD]	47.8 ± 12	49.7 ± 20	0.59^+^
TAPSE [mm]	20.9 ± 6.1	19.7 ± 5.2	0.33

**Table 1** Data are presented as n (%) or mean ± standard deviation. Unpaired t-test was applied, if not stated otherwise (^+^ = Welch-Test, ^C^ = Chi-Square-Test, ^F^ = Fisher's exact test). Abbreviations: ACE: angiotensin-converting enzyme. ALT: alanine transaminase. AT: Angiotensin. CTEPH: chronic thromboembolic pulmonary hypertension. ERA: endothelin receptor antagonist. FEV1: forced expiratory volume in 1 second. DLCO: diffusing capacity for carbon monoxide. FVC: forced vital capacity. Gamma-GT: Gamma Gamma-glutamyl transferase. Hs-CRP: high-sensitivity C-reactive protein. LDH: lactate dehydrogenase. LVEF: left ventricular ejection fraction. NT-proBNP: N-terminal prohormone of brain natriuretic peptide. PAH: pulmonary arterial hypertension. PDE5i: phosphodiesterase 5 inhibitor. PH: pulmonary hypertension. PH-CLD: pulmonary arterial hypertension in chronic lung disease. PH-LHD: pulmonary hypertension in left heart disease. Pred: predicted. Rtot: resistance. RV: residual volume. sGC: soluble guanylate cyclase. sPAP: systolic pulmonary artery pressure. TAPSE: tricuspid annular plane systolic excursion. TLC: total lung capacity. WHO: World Health Organization

**Table 2:** Iron status at baseline and follow-up in the total study population (n= 85)

	Intervention			Control		
	Baseline	Follow-up	p-value	Baseline	Follow-up	p-value
Haemoglobin [g/dl]	12.3 ± 2.3	12.7 ± 2.1	0.10	13.6 ± 2.0	13.3 ± 1.8	0.09
Haematocrit [%]	37.3 ± 6.8	38.2 ± 6.7	0.15	40.6 ± 5.7	39.5 ± 5.1	0.02*
Erythrocytes [x10^12^/L]	4.4 ± 0.8	4.4 ± 0.9	0.91	4.5 ± 0.7	4.4 ± 0.7	<0.01*
Serum iron [µg/dl]	43.2 ± 15.1	75.7 ± 34.4	<0.001*	102.9 ± 58.4	88.5 ± 40.0	0.05
Serum ferritin [ng/ml]	113.2 ± 239.1	255.7 ± 249.9	0.03*	114.1 ± 120.8	115.7 ± 123.2	0.87
Transferrin saturation [%]	10.8 ± 3.0	23.1 ± 12.2	<0.001*	27.0 ± 12.6	32.0 ± 50.3	0.51
Soluble transferrin receptor [mg/L]	2.8 ± 1.8	2.6 ± 1.7	0.61	1.7 ± 1.0	2.2 ± 1.6	0.02*

**Table 2**: Data are presented as mean ± standard deviation. * = significant. Paired t-test was applied.

**Table 3:** Laboratory assessment, pulmonary function testing, echocardiography, exercise capacity and symptomatology at baseline and follow-up, subdivided according to iron supplementation

	Intervention			Control		
	Baseline	Follow-up	p-value	Baseline	Follow-up	p-value
Laboratory assessment						
NT-proBNP [ng/l]	2448.5 ± 3811	2560.2 ± 4994	0.79	2647.3 ± 3835	2444.8 ± 3598	0.35
Creatinine [mg/dl]	1.3 ± 0.6	1.4 ± 0.9	0.49	1.2 ± 0.5	1.2 ± 0.5	0.46
Pulmonary function testing						
FEV1 [L]	1.6 ± 0.7	1.7 ± 0.7	0.02*	1.6 ± 0.7	2.8 ± 7.7	0.29
FVC [L]	2.1 ± 0.8	2.2 ± 0.7	0.04*	2.4 ± 0.9	2.5 ± 1	0.03*
TLC [L]	5.2 ± 1.6	4.9 ± 1.4	0.18	5.6 ± 1.7	5.7 ± 1.6	0.35
RV [L]	3.1 ± 1.4	2.8 ± 1.1	0.15	7.1 ± 26.3	3.6 ± 2.8	0.36
Rtot [% pred]	160.3 ± 119	146.7 ± 83	0.44	161.5 ± 166	149.7 ± 83.9	0.41
DLCO [% pred]	38.8 ± 16	41.4 ± 19	0.56	47.9 ± 19	49.3 ± 18.5	0.67
Echocardiography and electrocardiography						
Heart rate [n/min]	76.6 ± 16	73.4 ± 16.6	0.44	74.3 ± 13	76.4 ± 13.2	0.31
LVEF [%]	56.8 ± 13	57.7 ± 11	0.34	56.5 ± 15	55.1 ± 13	0.39
sPAP [mmHg + ZVD]	47.8 ± 12	45.4 ± 20	0.43	49.7 ± 20	45.9 ± 20.7	0.40
TAPSE [mm]	20.9 ± 6	20.1 ± 5.6	0.58	19.7 ± 5.2	19.9 ± 5.4	0.92
6-minute walk test						
Walk distance [m]	285.8 ± 124	300.2 ± 121	0.54	327.6 ± 137	337.5 ± 129	0.62
WHO function class						
			0.66			0.18^ C^
I	3 (9.7%)	4 (12.9%)		9 (16.7%)	9 (16.7%)	
II	13 (41.9%)	13 (41.9%)		22 (40.7%)	17 (31.5%)	
III	14 (45.2%)	13 (41.9%)		19 (35.2%)	22 (40.7%)	
IV	1 (3.2%)	1 (3.2%)		4 (7.4%)	6 (11.1%)	
Fatigue Score						
	5.6 ± 2.1	4.5 ± 2.0	0.01*	5.1 ± 2.7	5.4 ± 2.9	0.18

Table 3: Data are presented as mean ± standard deviation. * = significant. Paired t-test was applied, if not stated otherwise. ^C^ = Chi-Square-Test. Abbreviations: FEV1: forced expiratory volume in 1 second. DLCO: diffusing capacity for carbon monoxide. FVC: forced vital capacity. LVEF: left ventricular ejection fraction. PH: pulmonary hypertension. Pred: predicted. Rtot: resistance. RV: residual volume. sPAP: systolic pulmonary artery pressure. TAPSE: tricuspid annular plane systolic excursion. TLC: total lung capacity. TSH: thyroid-stimulation hormone. WHO: World Health Organization.
