# Supplementary material for: Intravenous iron supplementation in pulmonary hypertension groups 1 to 4
Source: Int J Med Sci. 2024 Aug 1;21(11):2011–20. doi: 10.7150/ijms.92904 (PMC11373552; doi:10.7150/ijms.92904)

Table 4: Baseline characteristics of group 1 PH patients (n = 30)

	Intervention (n = 8)	Control (n = 22)	p-value
Age [years]	62.8 ± 15.7	65.4 ± 14	0.66
Male	4 (50.0%)	9 (40.9%)	0.70^{C}
WHO function class			0.84^{F}
- I	1 (12.5 %)	4 (18.2 %)	
- II	4 (50.0 %)	12 (55.5 %)	
- III	3 (37.5 %)	6 (27.3 %)	
- IV	0 (0 %)	0 (0 %)	
Fatigue score	4.4 ± 1.9	4.9 ± 2.7	0.65
6-minute walk distance [m]	375.7 ± 141.3	330.2 ± 156.2	0.50
NT-proBNP [ng/l]	2544.8 ± 2041.1	1575.2 ± 2246.3	0.30
Haemoglobin [g/dl]	14.2 ±3.4	14.0 ± 2.6	0.81
Serum iron [µg/dl]	52.1 ± 17.3	117.1 ± 77.8	0.03*
Serum ferritin [ng/ml]	32.6 ± 31.2	83.9 ± 108.6	0.10
Transferrin saturation [%]	10.9± 2.7	29.0 ±15.7	<0.01*
Soluble transferrin receptor [mg/l]	2.1 ± 0.7	1.7 ± 0.9	0.62
sPAP [mmHg + ZVD]	55.1 ± 13	53.5 ±26	0.88
TAPSE [mm]	17.6 ± 6.5	19.8 ± 4.6	0.33

Table 4 Data are presented as n (%) or mean ± standard deviation. * = significant. Unpaired t-test was applied, if not stated otherwise (^{C} = Chi-Square-Test, ^{F} = Fisher's exact test). NT-proBNP: N-terminal pro-brain natriuretic peptide. PH: pulmonary hypertension. sPAP: systolic pulmonary artery pressure. TAPSE: tricuspid annular plane systolic excursion. WHO: World Health Organization

Table 5: Baseline characteristics of group 2 PH patients (n = 24)			
	Intervention (n = 9)	Control (n = 15)	p-value
Age [years]	75.7 ± 9.9	77.1 ± 8.0	0.70
Male	5 (55.5%)	10 (66.7%)	0.45[C]
WHO function class			0.37[F]
- I	0 (0.0 %)	4 (26.7 %)	
- II	3 (33.3 %)	3 (20.0 %)	
- III	5 (55.5 %)	6 (40.0 %)	
- IV	1 (11.1 %)	2 (13.3 %)	
Fatigue score	6.3 ± 1.8	5.5 ± 2.9	0.46
6-minute walk distance [m]	265.7 ± 133.5	236.4 ± 101.3	0.60
NT-proBNP [ng/l]	5316.2 ± 5565.6	4181.4 ± 5220.8	0.62
Haemoglobin [g/dl]	11.8 ± 0.9	13.6 ± 1.4	0.001*
Serum iron [µg/dl]	43.6 ± 11.11.6	90.8 ± 19.1	<0.001*
Serum ferritin [ng/ml]	219.2 ± 384.8	184.9 ± 187.9	0.77
Transferrin saturation [%]	12.8 ± 2.5	24.7 ± 7.6	<0.001*
Soluble transferrin receptor [mg/l]	4.7 ± 5.9	2.1 ± 1.4	0.11
sPAP [mmHg + ZVD]	47.2 ± 4.3	48.8 ± 13.7	0.73
TAPSE [mm]	18.0 ± 4.2	18.5 ± 5.6	0.82

Table 5 Data are presented as n (%) or mean ± standard deviation. * = significant. Unpaired t-test was applied, if not stated otherwise ([C] = Chi-Square-Test, [F] = Fisher's exact test). NT-proBNP: N-terminal pro-brain natriuretic peptide. PH: pulmonary hypertension. sPAP: systolic pulmonary artery pressure. TAPSE: tricuspid annular plane systolic excursion. WHO: World Health Organization

**Table 6: Baseline characteristics of group 3 PH patients (n = 22)**			
	Intervention (n = 10)	Control (n = 12)	p-value
Age [years]	67.1 ± 9.0	67.9 ± 9.3	0.84
Male	2 (20.0%)	3 (25.0%)	0.78[C]
WHO function class			0.43[F]
- I	1 (10.0 %)	1 (8.3 %)	
- II	4 (40.0 %)	6 (50.0 %)	
- III	5 (50.0 %)	3 (25.0 %)	
- IV	0 (0.0 %)	2 (16.7 %)	
Fatigue score	6.2 ± 2.5	5.1 ± 2.9	0.35
6-minute walk distance [m]	250.0 ± 117.9	318.1 ± 138.6	0.24
NT-proBNP [ng/l]	825.4 ± 979.2	569.5 ± 421.0	0.42
Haemoglobin [g/dl]	11.8 ± 1.2	13.5 ± 1.6	0.01*
Serum iron [µg/dl]	36.4 ± 14.8	111.0 ± 46.9	<0.001*
Serum ferritin [ng/ml]	116.7 ± 156.3	153.5 ± 126.8	0.55
Transferrin saturation [%]	10.5 ± 3.4	29.6 ± 10.7	<0.001*
Soluble transferrin receptor [mg/l]	2.7 ± 2.0	2.1 ± 1.4	0.43
sPAP [mmHg + ZVD]	40.1 ± 7.7	38.2 ± 12.1	0.68
TAPSE [mm]	25.0 ± 6.1	19.7 ± 3.9	0.04*

Table 6 Data are presented as n (%) or mean ± standard deviation. * = significant. Unpaired t-test was applied, if not stated otherwise ([C] = Chi-Square-Test, [F] = Fisher's exact test). NT-proBNP: N-terminal pro-brain natriuretic peptide. PH: pulmonary hypertension. sPAP: systolic pulmonary artery pressure. TAPSE: tricuspid annular plane systolic excursion. WHO: World Health Organization

Table 7: Baseline characteristics of group 4 PH patients (n = 9)			
	Intervention (n = 4)	Control (n = 5)	p-value
Age [years]	72.0 ± 3.7	76.4 ± 9.9	0.43
Male	2 (50.0%)	2 (40.0%)	0.76[C]
WHO function class			0.22[F]
- I	1 (25.0 %)	0 (0.0 %)	
- II	2 (50.0 %)	1 (20.0 %)	
- III	1 (25.0 %)	4 (80.0 %)	
- IV	0 (0.0 %)	0 (0.0 %)	
Fatigue score	4.8 ± 1.6	4.6 ± 2.3	0.91
6-minute walk distance [m]	300.0 ± 49.7	271.5 ± 122.9	0.68
NT-proBNP [ng/l]	1306.5 ± 1552.5	4260.6 ± 4289.4	0.24
Haemoglobin [g/dl]	11.5 ± 1.3	12.7 ± 0.9	0.14
Serum iron [µg/dl]	37.5 ± 9.8	73.8 ± 15.4	<0.01*
Serum ferritin [ng/ml]	16.9 ± 12.5	111.2 ± 119.5	0.17
Transferrin saturation [%]	8.8 ± 2.2	22.6 ± 4.8	0.001*
Soluble transferrin receptor [mg/l]	4.0 ± 3.4	1.2 ± 0.2	0.09
sPAP [mmHg + ZVD]	55.3 ± 22.5	61.9 ± 10.9	0.56
TAPSE [mm]	23.3 ± 2.1	22.4 ± 8.3	0.85

Table 7 Data are presented as n (%) or mean ± standard deviation. * = significant. Unpaired t-test was applied, if not stated otherwise ([C] = Chi-Square-Test, [F] = Fisher's exact test). NT-proBNP: N-terminal pro-brain natriuretic peptide. PH: pulmonary hypertension. sPAP: systolic pulmonary artery pressure. TAPSE: tricuspid annular plane systolic excursion. WHO: World Health Organization

Figure S1. Mean change of the SF-36 score domains from baseline to follow up, compared between intervention (i.v. iron supplementation) and controls for all patients and subgroups with PH 1-3. Notes: values represent mean ± standard deviation. P-values compare changes in controls and intervention group (Mann-Whitney U test). Asterisks (*) indicate a significant p-value <0.05 comparing the change of the respective SF-36 score between the two groups. + indicates a significant p-value <0.05 regarding change in the respective SF-36 score from baseline to follow-up within the intervention or control group. Abbreviations: SF-36: Short Form-36.

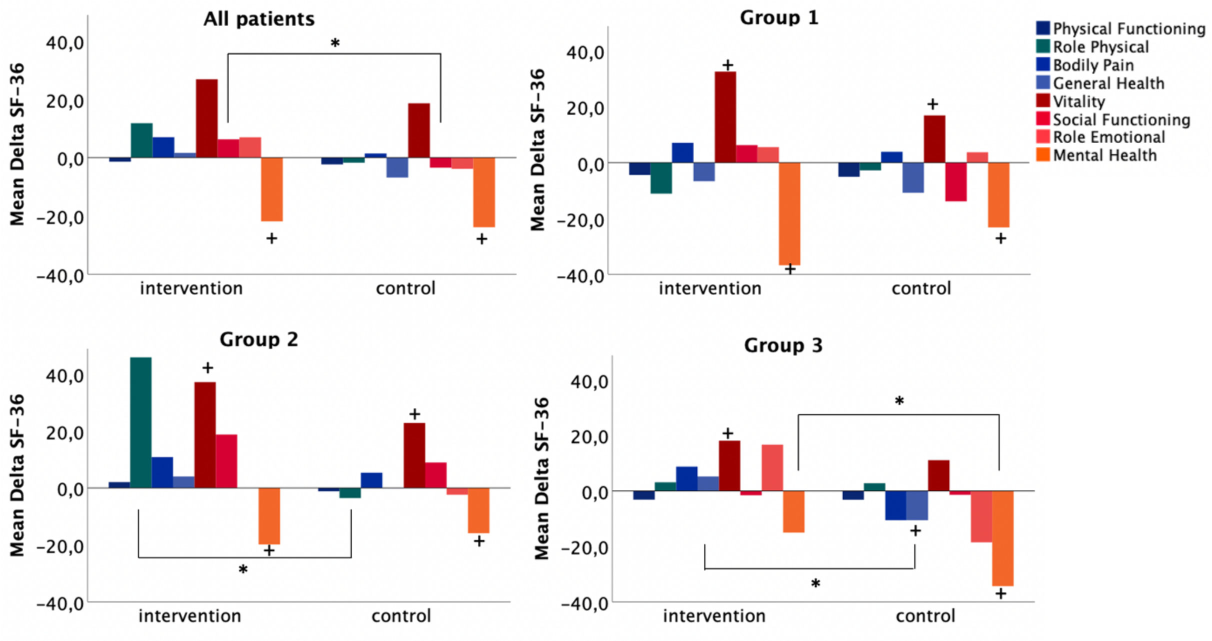

Supplement: Supplementary file 1 — Supplementary figures and tables. [file ijmsv21p2011s1.pdf]
